# Association of airway obstruction with first-pass success and intubation-related adverse events in the emergency department: multicenter prospective observational studies

**DOI:** 10.3389/fmed.2023.1199750

**Published:** 2023-05-25

**Authors:** Jin Takahashi, Tadahiro Goto, Shigeki Fujitani, Hiroshi Okamoto, Yusuke Hagiwara, Hiroko Watase, Kohei Hasegawa, the Japanese Emergency Medicine Network Investigators

**Affiliations:** ^1^Department of Emergency and Critical Care Medicine, Tokyo Bay Urayasu Ichikawa Medical Center, Urayasu, Chiba, Japan; ^2^Department of Emergency and Critical Care Medicine, St. Marianna University School of Medicine, Kawasaki, Kanagawa, Japan; ^3^TXP Medical Co., Ltd., Bunkyo, Tokyo, Japan; ^4^Department of Critical Care Medicine, St. Luke's International Hospital, Chuo-ku, Tokyo, Japan; ^5^Department of Pediatric Emergency and Critical Care Medicine, Tokyo Metropolitan Children’s Medical Center, Fuchu, Tokyo, Japan; ^6^Department of Emergency Medicine and General Internal Medicine, Fujita Health University School of Medicine, Toyoake, Aichi, Japan; ^7^Department of Emergency Medicine, Massachusetts General Hospital, Boston, MA, United States

**Keywords:** airway obstruction, first-pass success, intubation-related adverse events, emergency department, adults

## Abstract

**Background:**

Airway obstruction is a relatively rare but critical condition that requires urgent intervention in the emergency department (ED). The present study aimed to investigate the association of airway obstruction with first-pass success and intubation-related adverse events in the ED.

**Methods:**

We analyzed data from two prospective multicenter observational studies of ED airway management. We included adults (aged ≥18 years) who underwent tracheal intubation for non-trauma indications from 2012 through 2021 (113-month period). Outcome measures were first-pass success and intubation-related adverse events. We constructed a multivariable logistic regression model adjusting for age, sex, modified LEMON score (without airway obstruction), intubation methods, intubation devices, bougie use, intubator’s specialty, and ED visit year with accounting for patients clustering within the ED.

**Results:**

Of 7,349 eligible patients, 272 (4%) underwent tracheal intubation for airway obstruction. Overall, 74% of patients had first-pass success and 16% had intubation-related adverse events. The airway obstruction group had a lower first-pass success rate (63% vs. 74%; unadjusted odds ratio [OR], 0.63; 95% CI, 0.49–0.80), compared to the non-airway obstruction group. This association remained significant in the multivariable analysis (adjusted OR 0.60, 95%CI 0.46–0.80). The airway obstruction group also had a significantly higher risk of adverse events (28% vs. 16%; unadjusted OR, 1.93; 95% CI, 1.48–2.56, adjusted OR, 1.70; 95% CI, 1.27–2.29). In the sensitivity analysis using multiple imputation, the results remained consistent with the main results: the airway obstruction group had a significantly lower first-pass success rate (adjusted OR, 0.60; 95% CI, 0.48–0.76).

**Conclusion:**

Based on these multicenter prospective data, airway obstruction was associated with a significantly lower first-pass success rate and a higher intubation-related adverse event rate in the ED.

## Introduction

Tracheal intubation is a critical resuscitation procedure in the emergency department (ED). As multiple intubation attempts are associated with an increased risk of intubation-related adverse events (1–3), the early identification of patients at risk of intubation failure is essential. Airway obstruction is a relatively rare indication for airway management in EDs, with a reported incidence of 2–3% (1, 4, 5). Airway obstruction may lead to intubation failure—e.g., as “cannot intubate, cannot oxygenate” (CICO) situations, and cardiac arrest. Emergency physicians should aim to achieve first-pass success even in the challenging condition (6).

Despite the significance of airway obstruction in the EDs, little is known about the relationship between airway obstruction and intubation outcomes in the ED. Two small, single-center studies (*n* ≤ 366) evaluating factors associated with difficult intubation (i.e., the LEMON score) have found no association between airway obstruction and difficult intubation (7, 8). Further understanding of airway management in cases of airway obstruction and its relationship with intubation outcomes will facilitate the development of optimal strategies for ED airway management.

To address the major knowledge gap in the literature, we aimed to examine the association of airway obstruction with first-pass success and intubation-related adverse events by analyzing data of two multicenter prospective studies of ED patients.

## Methods

### Study design and setting

The present study a *post hoc* analysis data obtained from multicenter prospective observational studies of consecutive patients who underwent emergency airway management in EDs; the second and fifth Japanese Emergency Airway Network (JEAN-2 and -5) studies. The study design, setting, data collection methods, and measured variables of JEAN have been reported previously (1, 9–16). In summary, JEAN-2 comprised 15 academic and community EDs in various geographic regions across Japan and was conducted between February 2012 and March 2020. JEAN-5 is a subsequent study using standardized procedures that are similar to JEAN-2. JEAN-5 consisted of 14 EDs (accordingly, JEAN-2 and JEAN-5 comprised a total of 21 EDs) was conducted between April 2020 and July 2021. All EDs have emergency medicine residency programs and are staffed by emergency medicine attending physicians. Each ED has individual protocols for airway management. At the discretion of the attending physician, intubations are performed by attending physicians, resident physicians, or transitional-year residents (postgraduate years 1 and 2) rotating through EDs. The institutional review board of each participating institution approved the protocol with a waiver of informed consent before data collection.

### Data collection and processing

A standardized data collection form was completed by the intubator performing tracheal intubation immediately after each procedure. These data included patient demographics (age, sex, and estimated height and weight), the primary indication for intubation, intubation methods, administered medications, intubation devices, level of training and specialty of the intubator, number of intubation attempts, pre- and post-intubation vital signs, intubation success or failure, and intubation-related adverse events (14). The JEMNet (Japanese Emergency Medicine Network) Coordinating Centre and site investigator at each participating institution monitored compliance with data form completion. If there was any data missing from the data form, the data was returned to the intubator for completion. If there was any inconsistency in the data form, the intubator was contacted for clarification by the site investigator. We defined an intubation “attempt” as the insertion of a laryngoscope blade (or other devices) past the teeth (4, 14). An intubation attempt was defined as a success if a tracheal tube was passed through the vocal cord with placement confirmed by quantitative or colorimetric end-tidal carbon dioxide monitoring.

### Participant selection

The present analysis used data from adult (aged ≥18 years) patients who underwent tracheal intubation for non-trauma indications including cardiac arrest in EDs during a consecutive 113-month period (from February 2012 through July 2021). We excluded patients who underwent tracheal intubation for trauma and those with missing data, such as age, height, weight, intubation methods, intubation devices, or intubator’s specialty.

### Primary exposure

The primary exposure was airway obstruction as the primary indication (including airway obstruction caused by anaphylaxis but excluding that caused primarily by altered mental status). Other non-trauma indications for tracheal intubation (e.g., respiratory failure, shock, altered mental status, and cardiac arrest) were defined as no airway obstruction.

### Outcomes

The primary outcome of interest was first-pass success (2, 9, 13, 16, 17). The secondary outcome was overall intubation-related adverse events measured in EDs. Intubation-related adverse events were categorized into major (i.e., hypotension [systolic blood pressure < 90 mmHg], hypoxemia [pulse oximetry saturation < 90%], esophageal intubation with delayed recognition, cardiac arrest, and dysrhythmia) and minor (i.e., esophageal intubation with early recognition, endobronchial intubation, dental or lip trauma, regurgitation, and airway trauma) adverse events (1, 9, 15).

### Statistical analyses

First, we compared patient demographics, airway management characteristics, and intubation outcomes between the airway obstruction and the non-airway obstruction groups by using Mann–Whitney *U* test, Fisher’s exact, or the chi-square test as appropriate. Next, to determine the association between airway obstruction and each of the intubation outcomes, we constructed logistic regression models with generalized estimating equations to account for potential patients clustering within the ED. We also adjusted for potential confounders, including age (9, 15), sex, body mass index (BMI; <25.0, 25.0–29.9, and ≥30.0 kg/m^2^) (16), modified LEMON score (without airway obstruction), intubation methods (rapid sequence intubation, sedation without paralysis, no medication, and others), intubation devices (direct laryngoscopy, video laryngoscopy, fiberscope, and others) (13), bougie use (17), intubator’s specialty (transitional-year resident, emergency medicine resident, emergency attending physician, and other specialties), and ED visit year. As the modified LEMON criteria include airway obstruction, a score of 1 was given to modified LEMON if any criteria except airway obstruction were met. We selected these confounders based on clinical plausibility and *a priori* knowledge (10, 13, 15–17).

To determine the robustness of our inference, we conducted a series of sensitivity analyses. First, we calculated *E*-values and their lower 95% confidence interval (CI) limit. The E-value gauges the evidence for causality (18). The E-value indicates how strong an unmeasured confounder would have to be associated with both the exposure and outcome in order for the observed association not to be causal (18). For example, an *E*-value of 2.0 means that the OR for the associations of unmeasured confounders with both the exposure and outcome would have to be >2.0 to fully explain away the observed exposure-outcome association of interest. Second, there were because there were missing values for age, BMI, modified LEMON score, intubation devices, and intubator’s specialty, we imputed missing data using the multiple imputation method based on the assumption that missing was at random. All these variables and sex, intubation methods, bougie use, ED visit year, primary exposure (airway obstruction or no airway obstruction), and outcomes (first-pass success, overall, major, or minor adverse events) were used to predict imputation. We applied 20 imputed datasets using multivariable imputation using the chained equations (MICE) algorithm and then estimated odds ratios with 95% CI based on Rubin’s rules (19, 20). Third, we repeated the model using two additional outcomes: intubation success within two attempts, and rescue surgical airway attempts. *p*-values of <0.05 were considered statistically significant. Statistical analyses were performed using STATA 16.1 (StataCorp, College Station, TX) and JMP 14.0.0 (SAS Institute, Inc., Cary, NC).

## Results

During the 113-month study period, the JEAN-2 and JEAN-5 studies recorded a total of 14,312 patients who underwent emergency airway management in 21 EDs (capture rate, 97%; Supplementary Figure S1). Of these, the present study excluded 397 pediatric patients, 1,910 patients who underwent intubation for trauma, and 4,656 patients with missing data (age [*n* = 19], BMI [*n* = 844], modified LEMON score [*n* = 3,773], intubation devices [*n* = 6], and intubator’s specialty [*n* = 14]), leaving 7,349 patients with complete covariate data. Of these, 272 patients (4%) underwent tracheal intubation for airway obstruction.

Clinical and airway management characteristics are summarized in Table 1. Overall, the median age of study participants was 72 years (interquartile range [IQR], 60–81 years) and 62% of them were male. The airway obstruction group, compared to the non-airway obstruction group, comprised a greater proportion of obesity and modified LEMON score of ≥1 (both *p* < 0.05). In addition, patients with airway obstruction were less likely to have been intubated using a direct laryngoscope and more likely to have been intubated using a fiberscope or by an attending physician (all *p* < 0.05).

**Table 1:** Clinical and airway management characteristics of patients who underwent tracheal intubation in the emergency department according to airway obstruction.

	Airway obstruction *n* = 272 (4%)	No airway obstruction *n* = 7,077 (96%)	*P*-Value
Clinical characteristics			
Age, median (IQR), years	71 (55–81)	72 (60–81)	0.13
Male sex	165 (61)	4,360 (62)	0.75
Body mass index (kg/m^2^)^*^			
<25.0	194 (71)	5,453 (77)	0.03
25.0–29.9 (overweight)	55 (20)	1,240 (18)	0.25
≥30.0 (obesity)	23 (8)	384 (5)	0.03
Airway management characteristics			
Modified LEMON score ≥ 1 (without obstruction)^†^	140 (51)	2,938 (42)	0.001
Intubation methods^*^			
Rapid sequence intubation	82 (30)	2,584 (37)	0.03
Sedation without paralysis	101 (37)	653 (9)	<0.001
No medication	68 (25)	3,528 (50)	<0.001
Others	21 (7)	312 (4)	0.01
Intubation devices^*^			
Direct laryngoscope	122 (45)	4,172 (59)	<0.001
Video laryngoscope	114 (42)	2,865 (40)	0.64
Fiberscope	30 (11)	5 (<1)	<0.001
Others^‡^	6 (2)	35 (<1)	<0.001
Bougie use	15 (6)	107 (2)	<0.001
Intubator’s specialty^*^			
Transitional-year resident	80 (29)	2,868 (41)	<0.001
Emergency medicine resident	96 (35)	2,388 (34)	0.60
Emergency attending physician	64 (24)	1,265 (18)	0.02
Other specialties	32 (12)	556 (8)	0.02
ED visit year^*^			0.01
2012	24 (9)	556 (8)	
2013	26 (10)	986 (14)	
2014	34 (13)	940 (13)	
2015	30 (11)	862 (12)	
2016	44 (16)	949 (13)	
2017	27 (10)	825 (12)	
2018	27 (10)	517 (7)	
2019	29 (11)	432 (6)	
2020	21 (8)	565 (8)	
2021	10 (4)	445 (6)	

IQR, interquartile range. Data are shown as *n* (%) unless otherwise specified. ^*^Percentages may not equal 100 due to rounding. ^†^The modified LEMON score without airway obstruction consisted of (1) look externally, (2) interincisor distance, (3) hyoid-to-mental distance, and (4) neck mobility. ^‡^Defined as supraglottic device, nasal intubation and other devices.

**Figure 1:**
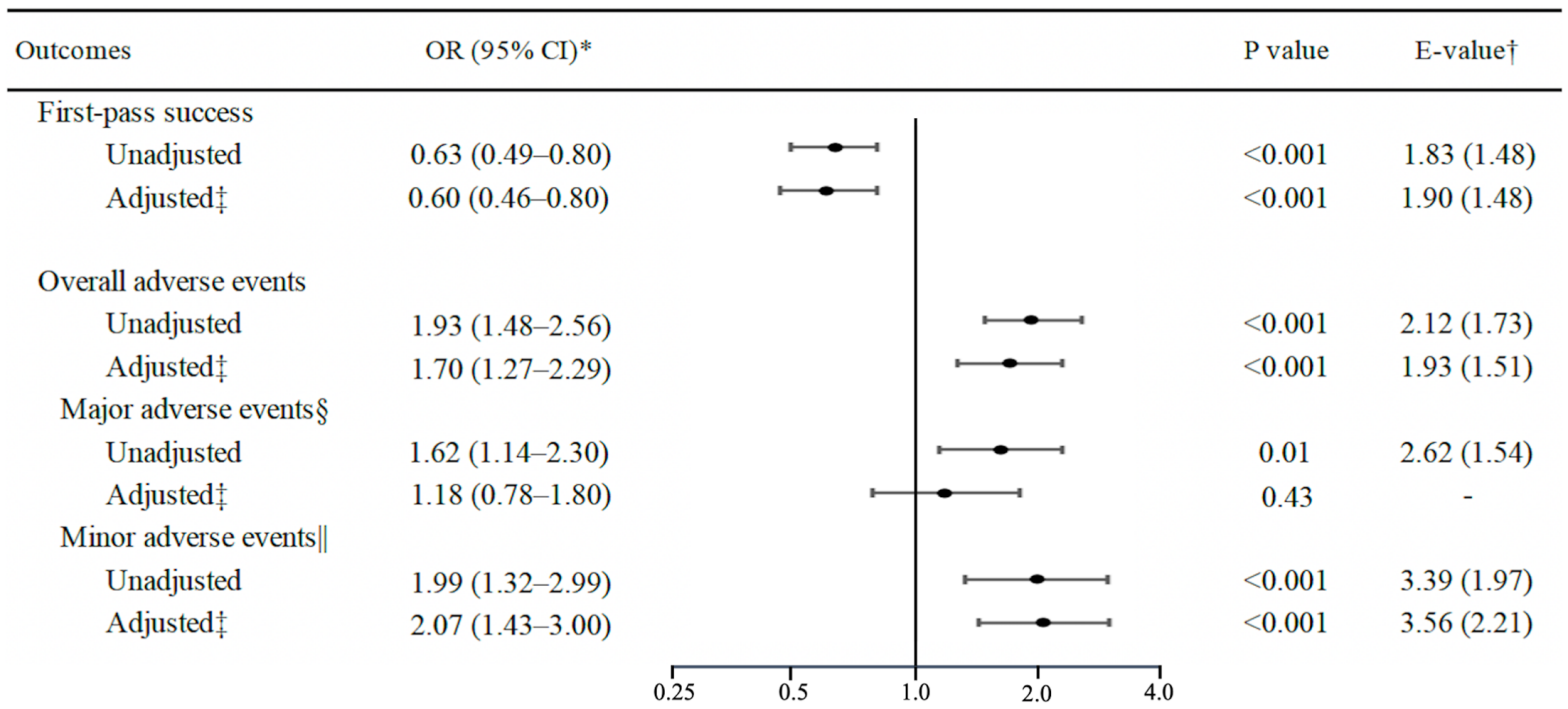
Unadjusted and adjusted associations of airway obstruction with first-pass success and intubation-related adverse events. ^*^Logistic regression models with generalized estimating equations were used to account for potential patients clustering within the emergency department. ^†^*E*-value (and its lower 95% CI limit) indicates the strength of the association between an unmeasured confounder(s) and both the exposure and outcome required to fully explain the observed association. ^‡^Adjusted for age, sex, body mass index, modified LEMON score, intubation methods, intubation devices, bougie use, intubator’s specialty, and ED visit year. ^§^Major adverse events included hypotension, hypoxemia, esophageal intubation with delayed recognition, cardiac arrest, and dysrhythmia. ^||^Minor adverse events included esophageal intubation with early recognition, endobronchial intubation, dental or lip trauma, regurgitation, and airway trauma. CI, confidence interval; OR, odds ratio.

Overall, the first-pass success rate was 74%. The airway obstruction group had a significantly lower first-pass success rate than the non-airway obstruction group (63% vs. 74%; unadjusted odds ratio [OR], 0.63; 95% CI, 0.49–0.80; *p* < 0.001; Table 2 and Figure 1). With adjusting for potential confounders, the association remained significant (adjusted OR, 0.60; 95%CI, 0.46–0.80; *p* < 0.001; *E*-value = 1.90). The overall rate of adverse events was 16% (major adverse events, 9%; minor adverse events, 9%) (Table 2). The airway obstruction group had significantly higher rates of overall (28% vs. 16%; *p* < 0.001), major (13% vs. 8%; *p* < 0.01), and minor (16% vs. 8%; *p* < 0.001) adverse events, compared to the non-airway obstruction group (Figure 1). Likewise, in the multivariable models, airway obstruction was associated with significantly higher rates of overall (adjusted OR, 1.70; 95% CI, 1.27–2.29; *p* < 0.001; *E*-value = 1.93) and minor (adjusted OR, 2.07; 95% CI, 1.43–3.00; *p* < 0.001; *E*-value = 3.56) adverse events.

**Table 2:** First intubation success and intubation-related adverse events according to airway obstruction.

Outcome	Airway obstruction *n* = 272 (4%)	No airway obstruction *n* = 7,077 (96%)	*P*-value^*^
**First-pass success**	171 (63)	5,231 (74)	<0.001
**Overall adverse events**	75 (28)	1,132 (16)	<0.001
**Major adverse events**	35 (13)	590 (8)	<0.01
Hypotension	19 (7)	354 (5)	0.14
Hypoxemia	18 (7)	245 (3)	0.01
Esophageal intubation with delayed recognition	3 (1)	16 (<1)	0.03
Cardiac arrest	0 (0)	21 (<1)	0.99
Dysrhythmia	1 (<1)	5 (<1)	0.20
**Minor adverse events**	43 (16)	601 (8)	<0.001
Esophageal intubation with early recognition	17 (6)	239 (3)	0.02
Endobronchial intubation	7 (3)	158 (3)	0.54
Dental or lip trauma	8 (3)	169 (2)	0.17
Regurgitation	8 (3)	91 (1)	0.03
Airway trauma	7 (3)	22 (<1)	<0.001

Data are shown as *n* (%) unless otherwise specified. ^*^Outcomes were compared using Fisher’s exact test or chi-square test as appropriate.

**Figure 2:**
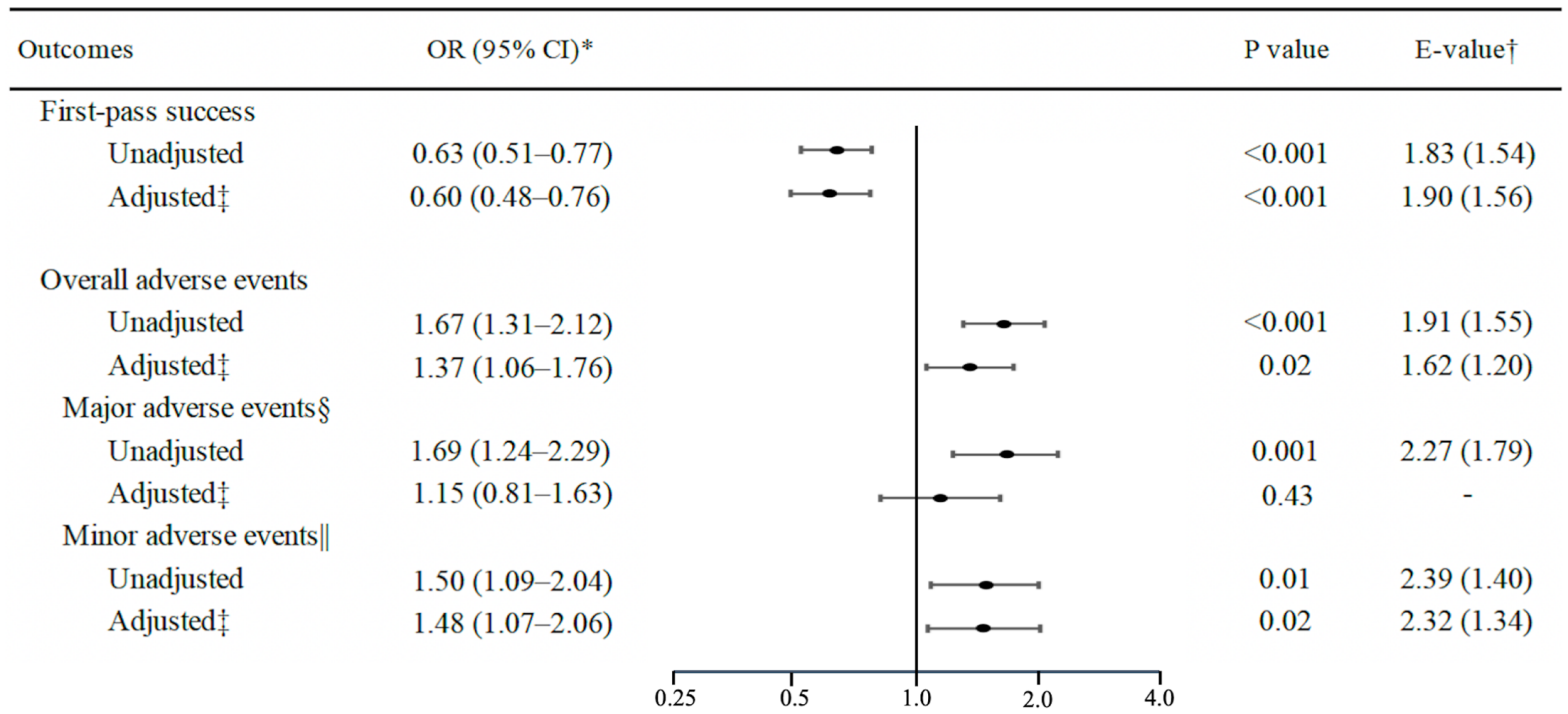
Unadjusted and adjusted associations of airway obstruction with first-pass success and intubation-related adverse events using multiple imputation. ^*^Logistic regression models with generalized estimating equations were used to account for potential patients clustering within the emergency department. ^†^*E*-value (and its lower 95% CI limit) indicates the strength of the associations between an unmeasured confounder(s) and both the exposure and outcome required to fully explain the observed associations. ^‡^Adjusted for age, sex, body mass index, modified LEMON score, intubation methods, intubation devices, bougie use, intubator’s specialty, and ED visit year. ^§^Major adverse events included hypotension, hypoxemia, esophageal intubation with delayed recognition, cardiac arrest, and dysrhythmia. ^||^Minor adverse events included esophageal intubation with early recognition, endobronchial intubation, dental or lip trauma, regurgitation, and airway trauma. OR, odds ratio; CI, confidence interval.

In the sensitivity analyses, first, in the analysis using multiple imputation, the results remained consistent with the main results — e.g., patients with airway obstruction had a significantly lower first-pass success rate (adjusted OR, 0.60; 95% CI, 0.48–0.76; *p* < 0.001; *E*-value = 1.90; Figure 2). Second, airway obstruction was significantly associated with a lower rate of intubation success within two attempts (OR, 0.58; 95% CI, 0.40–0.85; *p* < 0.01; *E*-value = 1.95) and higher rate of rescue surgical airway attempt (unadjusted OR, 8.91; 95% CI, 3.35–23.74; *p* < 0.001; Table 3).

**Table 3:** Unadjusted and adjusted associations of airway obstruction with intubation success within two attempts and rescue surgical airway attempt.

Outcome	Airway obstruction *n* = 272 (4%)	No airway obstruction *n* = 7,077 (96%)	Unadjusted OR^*^ (95% CI)	*P*-value	*E*-value^†^	Adjusted OR^*‡^ (95% CI)	*P*-value	*E*-value^†^
Intubation success within two attempts	232 (85)	6,500 (92)	0.54 (0.38–0.77)	<0.001	2.06 (1.54)	0.58 (0.40–0.85)	<0.01	1.95 (1.39)
Rescue surgical airway attempt^*^	6 (2)	19 (<1)	8.91 (3.35–23.74)	<0.001	17.31 (6.16)	N/A§	N/A^§^	N/A^§^

Data are shown as n (%) unless otherwise specified. Abbreviations: CI, confidence interval; N/A, not applicable; OR, odds ratio. ^*^Logistic regression models with generalized estimating equations were used to account for potential patients clustering within emergency department. ^†^*E*-value (and its lower 95% CI limit) indicates the strength of the associations between an unmeasured confounder(s) and both the exposure and outcome required to fully explain the observed associations. ^‡^Adjusted for age, sex, body mass index, modified LEMON score, intubation methods, intubation devices, bougie use, intubator’s specialty, and ED visit year. ^§^The value for this variable could not be estimated due to the small number of events.

## Discussion

In the present analysis of data from two multicenter prospective studies of 7,349 ED intubations, airway obstruction was associated with a significantly lower first-pass success rate and a significantly higher risk of intubation-related adverse events. The observed associations were consistent across the different analytical assumptions. Patients with airway obstruction also had a significantly lower rate of intubation success within two attempts and a higher rate of rescue surgical airway attempts. To the best of our knowledge, this is the first investigation that has demonstrated the relationship of airway obstruction with worse intubation outcomes in the ED.

The sparse ED literature on this research topic makes direct comparison with our observations difficult. In the field of anesthesiology, 40% of major intubation-related adverse events (e.g., death) in the operation room were due to airway abnormalities involving the head, neck, and trachea, and 70% of those were due to airway obstruction (21). In the ED setting, a descriptive study of intubation for angioedema reported a first-pass success rate of 81% (22). Potential reasons for the high first-pass success rate include the more-frequent use of the fiberscope (49%) and potential underestimation of intubation attempt frequency (22). In contrast to our findings, two smaller single-center studies (*n* ≤ 366) investigating predictors of intubation difficulty found no significant association between airway obstruction and intubation difficulty (7, 8). The discrepancy between the findings may be attributable to the difference in the study design, sample, data collection measures, study sample size, or any combination of these factors. Regardless, our study—with a sample size many times larger—build on these earlier reports and extend them by demonstrating the relationship between airway obstruction and intubation outcomes in the ED.

The underlying mechanisms of the observed association are likely multifactorial. First, the underlying cause(s) of airway obstruction may further hinder visualization of the vocal cords and tracheal intubation. Poor visualization of the vocal cord is known to increase the technical difficulty of intubation (23). Indeed, in the present study, esophageal intubation (with early or delayed recognition), airway trauma, and hypoxemia were more frequently observed in the airway obstruction group, supporting this mechanism (23). Second, as each patient has a different (partial or complete) level of airway obstruction, the optimal intubation methods and devices are different between patients (6, 24). This complexity and cognitive load might have led to intubation failure and adverse events (24). Lastly, these mechanisms are not mutually exclusive. Regardless of the complexity of these potential mechanisms, emergency physicians should develop optimal airway management strategies and technical skills (e.g., fiberscope, bougie, and surgical airway procedures) for patients with airway obstruction. In addition to these technical improvements, individual or team simulation training and *in-situ* supervision by attending physicians may increase the effectiveness of airway management (25, 26).

The present study has several potential limitations. First, airway obstruction was primarily determined by the judgment of the intubator as the study does not have data on the actual causes of airway obstruction. This might have introduced information bias. Second, we excluded traumatic airway obstruction because medical and traumatic airway obstruction cannot be dealt with as the same due to their differences in physiological and anatomical factors. In addition, JEAN registries did not collect information on whether pharyngeal foreign bodies were removed with Magill forceps. In cases where intubation was necessary due to airway obstruction caused by a foreign body, we assumed that the removal of the foreign body and intubation was considered a single event. Regardless, the rate of airway obstruction observed in the current study was 2.9%, which was consistent with that of earlier reports (i.e., 1.7–3.2%) (4, 5). Third, the exclusion of patients with a missing value may have introduced selection bias. To address this issue, we performed a subsequent analysis using multiple imputation, which demonstrated consistent inference. Fourth, self-reporting bias may have overestimated the first-pass success rate and underestimated the rate of adverse events. However, the study was based on a previously defined self-reporting system with standardized data forms and a high capture rate (97%). Fifth, the causal inference may be confounded by unmeasurable confounders, such as patient demographics (e.g., underlying diseases) and factors related to airway obstruction (e.g., malformation or obstruction severity). Nevertheless, the E-values supported the robustness of our inference. Finally, the generalizability of our findings may be limited in other ED settings. Nevertheless, the observed relationships are plausible.

## Conclusion

On the basis of data from two multicenter prospective studies of 7,349 patients who underwent tracheal intubation, patients with airway obstruction had a significantly lower first-pass success rate and a significantly higher rate of intubation-related adverse events. For clinicians, our data underscore the importance of identifying these high-risk patients and the systematic use of rescue intubation techniques in emergency airway management. Furthermore, our observations should facilitate further investigation into the optimal airway management practice in the ED, which will, in turn, lead to better outcomes in critically-ill patients.

## Data availability statement

IRB does not allow data sharing.

## Ethics statement

The protocols for the studies were approved by the Ethics Committees of Tokyo Bay Urayasu Ichikawa Medical Center (approval number of JEAN-2 873, approval number of JEAN-5 533). Written informed consent for participation was not required for these studies in accordance with the institutional requirements.

## Author contributions

JT took responsibility for the manuscript as a whole. JT, TG, SF, and KH conceived the study. HO, YH, HW, and KH supervised the conduct of the study. TG, SF, and KH provided the statistical advice. JT and TG performed the data analyses. JT drafted the manuscript. All authors contributed substantially to manuscript revision.

## Funding

The present study was supported by grants from St. Luke’s Science Institute (H26, H27) (Tokyo, Japan). The study sponsor had no involvement in the study design, data collection, data analysis and interpretation, manuscript preparation, or in the decision to submit the manuscript for publication.

## Conflict of interest

TG was employed by TXP Medical Co., Ltd.

The remaining authors declare that the research was conducted in the absence of any commercial or financial relationships that could be construed as a potential conflict of interest.

## Publisher’s note

All claims expressed in this article are solely those of the authors and do not necessarily represent those of their affiliated organizations, or those of the publisher, the editors and the reviewers. Any product that may be evaluated in this article, or claim that may be made by its manufacturer, is not guaranteed or endorsed by the publisher.
