# Corrigendum: Association of airway obstruction with first-pass success and intubation-related adverse events in the emergency department: multicenter prospective observational studies

**DOI:** 10.3389/fmed.2023.1307868

**Published:** 2023-10-20

**Authors:** Jin Takahashi, Tadahiro Goto, Shigeki Fujitani, Hiroshi Okamoto, Yusuke Hagiwara, Hiroko Watase, Kohei Hasegawa, The Japanese Emergency Medicine Network Investigators

**Affiliations:** ^1^Department of Emergency and Critical Care Medicine, Tokyo Bay Urayasu Ichikawa Medical Center, Urayasu, Chiba, Japan; ^2^Department of Emergency and Critical Care Medicine, St. Marianna University School of Medicine, Kawasaki, Kanagawa, Japan; ^3^TXP Medical Co., Ltd., Bunkyo, Tokyo, Japan; ^4^Department of Critical Care Medicine, St. Luke's International Hospital, Chuo-ku, Tokyo, Japan; ^5^Department of Pediatric Emergency and Critical Care Medicine, Tokyo Metropolitan Children's Medical Center, Fuchu, Tokyo, Japan; ^6^Department of Emergency Medicine and General Internal Medicine, Fujita Health University School of Medicine, Toyoake, Aichi, Japan; ^7^Department of Emergency Medicine, Massachusetts General Hospital, Boston, MA, United States

**Keywords:** airway obstruction, first-pass success, intubation-related adverse events, emergency department, adults

In the published article, there was an error in the Ethics statement. The registries' institutional review boards of Kurashiki Hospital and St. Luke's Hospital were listed, not the institutional review board to which the first author belongs. “The protocols for the studies were approved by the Ethics Committees of Kurashiki Central Hospital (approval number 851-2) and St. Luke's International Hospital (approval number 19-R212). Written informed consent for participation was not required for these studies in accordance with the institutional requirements.” The correct Ethics statement appears below.

“The protocols for the studies were approved by the Ethics Committees of Tokyo Bay Urayasu Ichikawa Medical Center (approval number of JEAN-2 873, approval number of JEAN-5 533). Written informed consent for participation was not required for these studies in accordance with the institutional requirements.”

The authors apologize for this error and state that this does not change the scientific conclusions of the article in any way. The original article has been updated.

